# Identification and Validation of Autophagy-Related Genes in Necrotizing Enterocolitis

**DOI:** 10.3389/fped.2022.839110

**Published:** 2022-04-28

**Authors:** Yuxin Tian, Mengjia Mao, Xuqing Cao, Haitao Zhu, Chun Shen

**Affiliations:** Department of Pediatric Surgery, Children's Hospital of Fudan University, National Children's Medical Center, Shanghai, China

**Keywords:** necrotizing enterocolitis, autophagy, bioinformatics analysis, hypoxia, HIF-1a

## Abstract

**Background:**

Autophagy plays an essential role in the occurrence and progression of necrotizing enterocolitis (NEC). We intend to carry out the identification and validation of the probable autophagy-related genes of NEC *via* bioinformatics methods and experiment trials.

**Methods:**

The autophagy-related differentially expressed genes (arDEGs) of NEC were identified by analyzing the RNA sequencing data of the experiment neonatal mouse model and dataset GSE46619. Protein–protein interactions (PPIs), Gene Ontology (GO) enrichment analysis, and Kyoto Encyclopedia of Genes and Genomes (KEGG) pathway enrichment analysis were used for the arDEGs. Then, co-expressed autophagy-related genes in two datasets were identified by Venn analysis and verified by qRT-PCR in experimental NEC.

**Results:**

Autophagy increased in experimental NEC and 47 arDEGs were identified in experimental NEC by RNA-sequencing. The PPI results proclaimed those genes interplayed with each other. The GO and KEGG enrichment results of arDEGs reported certain enriched pathways related to autophagy and macroautophagy. Furthermore, 22 arDEGs were identified in human NEC from dataset GSE46619. The GO and KEGG enrichment analysis of these genes showed similar enriched terms with the results of experimental NEC. Finally, HIF-1a, VEGFA, ITGA3, ITGA6, ITGB4, and NAMPT were identified as co-expressed autophagy-related genes by Venn analysis in human NEC from dataset GSE46619 and experimental NEC. The result of quantified real-time PCR (qRT-PCR) revealed that the expression levels of HIF-1a and ITGA3 were upregulated, while VEGFA and ITGB4 were downregulated in experimental NEC.

**Conclusion:**

We identified 47 arDEGs in experimental NEC and 22 arDEGs in human NEC *via* bioinformatics analysis. HIF-1a, ITGA3, VEGFA, and ITGB4 may have effects on the progression of NEC through modulating autophagy.

## Introduction

Necrotizing enterocolitis (NEC) is considered one of the most severe gastrointestinal emergencies in neonatal surgery, with a prevalence of up to 7% and an estimated rate of death between 30 and 50% in preterm infants (1). Neonatal NEC still requires surgical intervention, for which effective clinical management is currently limited, and long-term complications of NEC are even related to an increased risk for neurodevelopmental delay (2, 3). The major risk factors consist of intestine prematurity, formula feeding, bacterial colonization, and perinatal asphyxia, but the clear mechanism of NEC is not completely understood (1, 4).

In general, autophagy is a highly conserved catabolic process that generally protects cells from various stressors (5, 6). Autophagy is able to conduct cellular homeostasis, but undisciplined activation of autophagy may cause cellular damage and cell death (7, 8). Various conditions of stress can activate autophagy, such as nutrient deprivation, hypoxia, oxidative stress, and endoplasmic reticulum stress (9–14). Autophagosome formation is the marker of autophagy initiation and is mediated by the ULK1 complex, which interacts with the phosphoinositide 3-kinase catalytic subunit type III (PI3KC3) complex to form phagocytic vesicles (15). Beclin1, another marker of autophagy, is a segment of the type III PI3-kinase complex which is involved in the autophagic vesicle's nucleation. Beclin1 is necessary for the installation and initiation of autophagosome construction through recruiting autophagy-related proteins to the pre-autophagosomal structure (6). Then, the ATG12-ATG5-ATG16 complex promotes the conjugating LC3 to phosphatidylethanolamine. In this conjugation reaction, the lipid form of LC3I is converted to the autophagosome-associated form LC3II, and this alteration is applied as a hallmark of autophagy (6). Finally, the contents of the autophagosome are degraded after the interactions between the autophagosome and a lysosome. p62 (also known as SQSTM1) is a well-known autophagy substrate. It is necessary for p62 protein to interact with LC3 during the process of autophagosome formation, and besides, the degradation of p62 protein is the hallmark of autophagy activation.

In addition to its role in normal physiology, autophagy plays a critical role in pathological processes, such as cancer, infections, neurodegeneration, aging, tuberculosis, and heart disease (7, 9, 12–14). Recently, autophagy has been linked to inflammatory bowel disease (IBD). Moreover, autophagy has also been reported to be activated respectively in the intestine tissues of patients with NEC and the ileum tissues of NEC rats (16). However, autophagy-related genes in NEC are unrecognized and thus require deeper exploration. Exploring and validating the possible autophagy-related genes in NEC will provide us with the latent biomarkers of NEC clinical treatment.

Here, we reported the arDEGs of NEC by analyzing the RNA-sequencing data of the mouse model and dataset GSE46619 from GEO databases. Moreover, protein–protein interaction (PPI) analysis, Gene Ontology (GO), and Kyoto Encyclopedia of Genes and Genomes (KEGG) pathway enrichment analysis were administered for the arDEGs. Consequently, 6 pivotal DEGs were identified and further verified in the mouse model of NEC.

## Materials and Methods

### Ethics Statement

All animal trials were approved by the Institutional Animal Ethics Committee of Children's Hospital of Fudan University and performed in compliance with the national and international guidelines for the Care and Use of Laboratory Animals.

### NEC Mouse Model

Neonatal C57BL/6 pups from 5- to 9-day-old were used to induce an experimental NEC model. The induction of NEC involves gavage feeding of hyperosmolar formula milk (15 g Similac and 75 ml Esbilac; 50 μl/kg body weight per day), exposure to hypoxia (5% O_2_ for 10 min and 3 times/day) for 4 days, and lipopolysaccharide (LPS) stimulation (4 mg/kg/day) with oral feeding on postnatal days 6 and 7. Pups were randomly assigned to the control group (CTRL group) and experimental group (NEC group). All mice were harvested 3 h after the last hypoxia on postnatal day 9 and the intestine tissues were collected separately.

### Hematoxylin-Eosin (H&E) Staining

The terminal ileum tissues were fixed overnight in 4% paraformaldehyde, embedded in paraffin, and cut into 3 μm thick sections. Then, the prepared sections were stained using hematoxylin and 3% eosin separately. Based on a modified NEC scoring system according to a previous study (17), a histopathological examination was performed by two independent pathologists with no prior knowledge of the mouse group. The NEC scoring system was as follows: Score 0: normal; Score 1: mild to moderate submucosal separation and/or submucosal and muscular layer edema; Score 2: severe submucosal separation and/or submucosal and muscular layer edema with the localized loss of villi; and Score 3: loss of intestinal villi with intestinal necrosis. Pups with histological scores ≥2 were defined as the presence of NEC injury. The histopathological condition of the terminal ileum tissues from pups in each group was observed and noted with photographs.

### Gene Expression

RNA was isolated from intestinal tissue using TRIzol (Invitrogen, Carlsbad, CA) according to the manufacturer's instructions. TB Green® Premix Ex Taq™ II (TAKARA, Japan) was used for quantitative real-time PCR (RT-PCR) to measure the expression levels of inflammatory markers interleukin-6 (IL-6) and stem cell marker Leucine-rich repeat-containing G-protein coupled receptor 5 (Lgr5) for the validation of experimental NEC mouse model. Results were generated from three independent experiments with each carried out in technical triplicate. Relative gene expression levels were calculated by the ΔΔCt method and standardized to the reference housekeeping gene GAPDH. Forward and reverse primers were designed and verified using the National Center for Biotechnology Information (NCBI) Primer-BLAST online program as shown in Supplementary Table 1.

### Immunohistochemistry (IHC)

Slides were deparaffinized in 100% xylene three times (5 min each), rehydrated through stepwise washing in a down-trend ethanol solution ratio (100, 95, 80, and 75%) for 5 min each at room temperature (RT), and then treated with 3% hydrogen peroxide for 15 min. Antigen retrieval was implemented by boiling slides in 0.01 M sodium citrate buffer (pH 6.0) in the autoclave (80 kpa and 100°C) for 3 min, and then cooling to RT. Sections were incubated with 10% BSA (Sigma, Germany) for 1 h to block nonspecific antigens and with primary antibodies overnight at 4°C, followed by incubation with the components of the Envision-plus detection system (EnVision +/HRP/Mo, Dako) for 1 h. The sections were stained with 3,3'-diaminobenzidine for 1 min and counterstained with hematoxylin for 1 min at RT. Negative controls were treated identically except without adding the primary antibodies. The final staining score was ranked in four grades according to the values of immunoreaction intensity and percentage of cell staining: Score 0: weak, <25% value; Score 1: moderate, between 25 and 50% value; Score 2: strong, between 50 and 75% value; and Score 3: very strong, higher than 75% value.

### RNA Library Construction and Sequencing

Total RNA was extracted using the RNeasy mini kit (Qiagen, Germany), and the RNA concentration and quality were performed using the Qubit®2.0 Fluorometer (Life Technologies, USA) and the Nanodrop One spectrophotometer (Thermo Fisher Scientific Inc., USA). The integrity of total RNA was tested using the Agilent 2100 Bioanalyzer (Agilent Technologies Inc., USA), and samples (RNA integrity number >7) were used for the following sequencing. RNA-seq strand-specific libraries were set up using the VAHTS Total RNA-seq (H/M/R) Library Prep Kit (Vazyme, China) according to the manufacturer's instructions. After removing rRNA, RNA was purified and fragmented into small pieces using divalent cations for 8 min at 94°C, followed by copying into cDNA using reverse transcriptase, DNA polymerase I, and RNase H. Those products were purified and enriched by PCR to build up the cDNA library which was quantified and verified by the Qubit® 2.0 Fluorometer and Agilent 2100 bioanalyzer. The cluster was produced by cBot after the library was diluted to 10 pM and then was sequenced on the Illumina NovaSeq 6000 platform (Illumina, USA).

### Autophagy-Related Genes Datasets and Microarray Data

A total of 222 genes were collected from The Human Autophagy Database (http://www.autophagy.lu/index.html). The mRNA expression profile dataset of GSE46619 was downloaded from the Gene Expression Omnibus (GEO) database (http://www.ncbi.nlm.nih.gov/geo/). GSE46619, from GPL6244 platform (Affymetrix Human Gene 1.0 ST Array [transcript (gene) version]), included 5 NEC and 4 normal human small intestinal tissue samples.

### Differentially Expressed Analysis of Autophagy-Related Genes

The normalized expression matrix of microarray data was downloaded from the GSE38974 dataset and annotated by the annotation files. Then, arDEGs were identified by R software with an adjusted *p* < 0.05 and absolute fold-change value >2. The heatmap, volcano plot, and box plot were performed with R software.

### PPI Analysis and Correlation Analysis of the arDEGs

Protein-protein analysis of those differentially expressed autophagy-related genes was carried out using the STRING database (https://string-db.org/) and Cytoscape software (version 3.8.1). Then, the correlation analysis was performed using Spearman's correlation in the “corrplot” package of R software.

### GO and KEGG Pathway Enrichment

Gene Ontology and KEGG pathway enrichment analysis were carried out using the package “GO plot” of R software. The GO analysis involved cellular component (CC), biological process (BP), and molecular function (MF), respectively.

### Statistical Analysis

The statistical analysis was performed using R software (version 3.6.1) and Prism (version 8.0). Normally distributed measurement data are explicated as mean ± standard deviation (mean ± SD), using an unpaired *t*-test for comparison between two groups. Count data are expressed as median (interquartile spacing). A *p* lower than 0.05 was considered statistically significant.

## Results

### Establishment and Validation of Mouse Model of NEC

**Figure 1:**
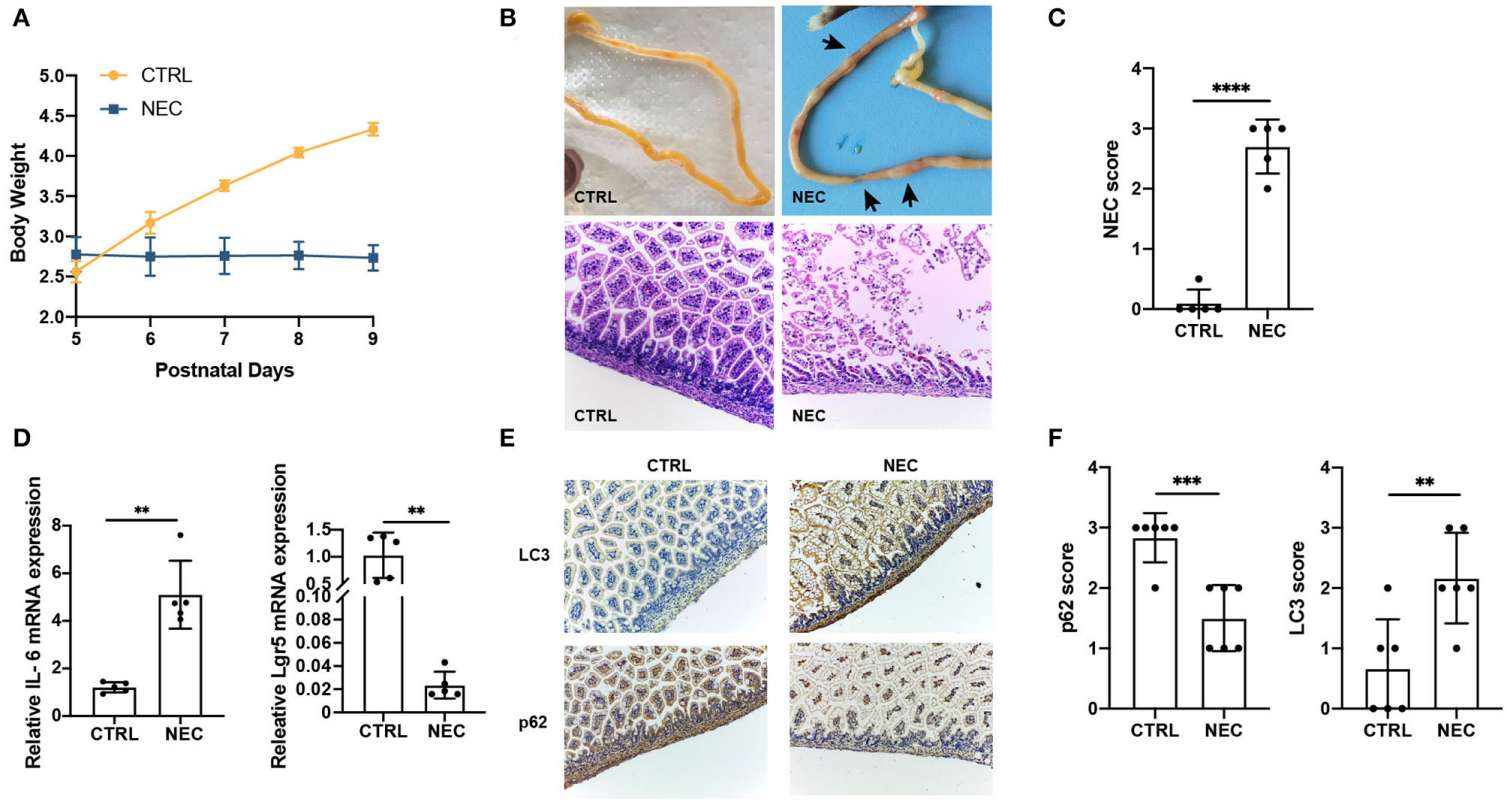
Validation of the necrotizing enterocolitis (NEC) model and upregulated the autophagy level. **(A)** Changes in the bodyweight of mice in NEC and CTRL groups. **(B)** The representative images of morphology and H and E micrographs of the distal ileum of NEC and CTRL mice. **(C)** The NEC scores in NEC and CTRL groups. **(D)** The relative interleukin 6 (IL-6) and Leucine-rich repeat-containing G-protein coupled receptor 5 (Lgr5) messenger RNA (mRNA) expression in NEC and CTRL groups. **(E)** Representative immunohistochemistry (IHC) staining of LC3 and p62 in ileum tissues of NEC and CTRL mice. **(F)** Immunohistochemistry scores of LC3 and p62 in NEC and CTRL groups. Data are expressed as mean ± SD. **p < 0.01; ***p < 0.001; ****p < 0.0001.

The NEC mouse model was induced by hypoxia, oral LPS, and artificial feeding with hyperosmolar formula milk. The bodyweight of mother-fed pups (CTRL group) rose faster with a good nutritional status, while that of the NEC group basically stayed the same on a postnatal day 5 (Figure 1A). The gross appearance of the intestine has good elasticity and continuity in the control group, while typical NEC-like injuries can be seen in the NEC group, such as expansion, gas accumulation, and even intestinal hemorrhage. The images of the intestines in both groups are shown in Figure 1B. The double-blind NEC score of the NEC group was obviously higher than that of the control group which is shown in Figure 1C. In intestinal sections from control pups, we observed that the morphology of the intestinal villi was intact with no morphologic change; while in the sections from NEC pups, the villi structure was disrupted dramatically (Figure 1B). Compared with the CTRL group, there was a significant upregulation of inflammatory cytokine IL-6 and downregulation of intestinal stem cell marker Lgr5 in the NEC group (Figure 1D). Those results suggested that the animal model of NEC was successfully established.

To investigate whether autophagy was associated with NEC, the markers of autophagy, LC3, and p62, were examined in the NEC and CTRL groups by IHC staining. Compared with CTRL groups, LC3 expression was significantly upregulated, and p62 expression was downregulated in ileum sections from NEC groups (Figures 1E,F). Together, these results demonstrated autophagy increased in NEC mice compared with CTRL mice.

### Identification of arDEGs in a Mouse Model of NEC

**Figure 2:**
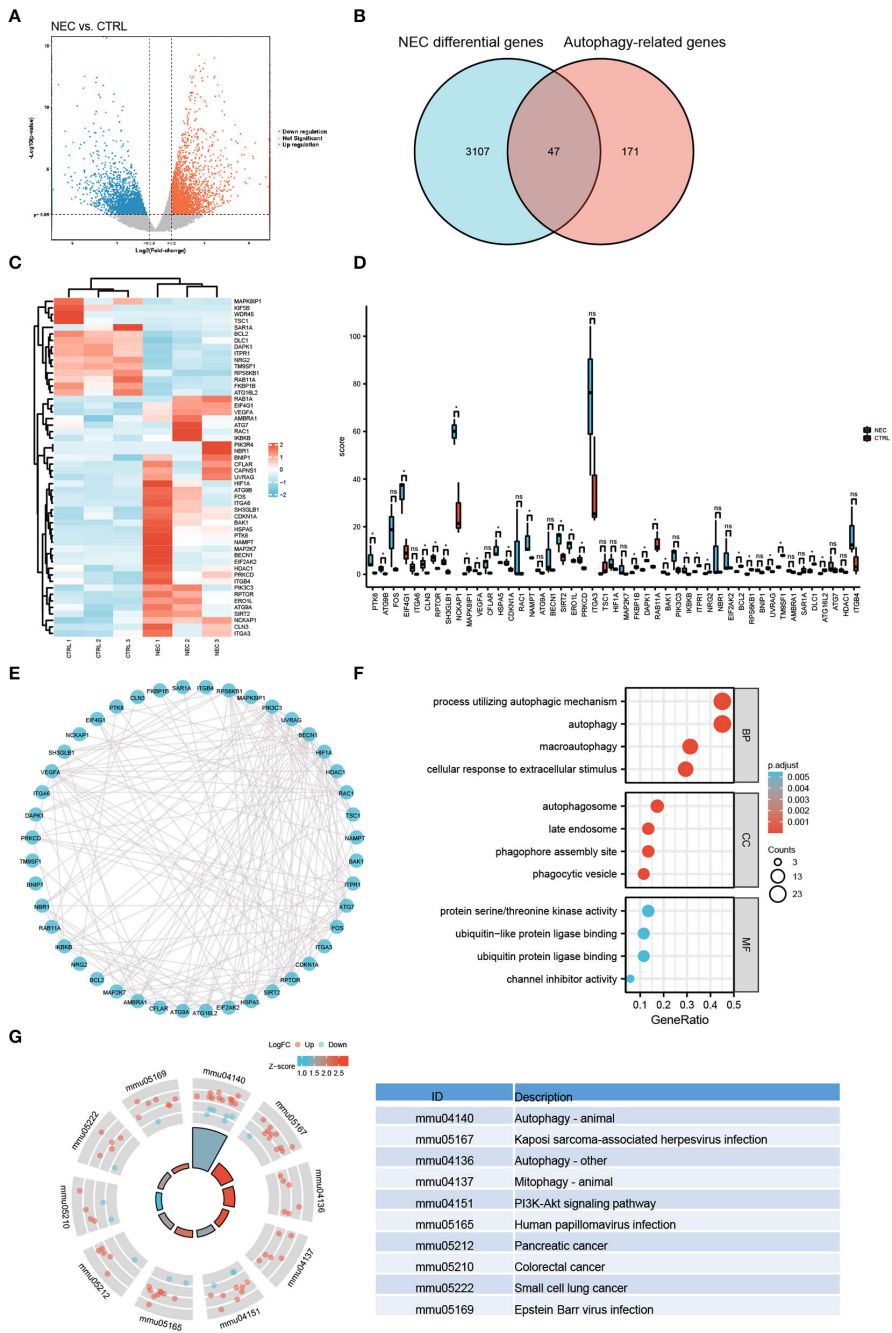
Autophagy-related differentially expressed genes (arDEGs) autophagy-related genes in our neonatal mouse model of NEC. **(A)** Volcano plot of the differentially expressed genes (DEGs) in the NEC group. The red dots represent the significantly upregulated genes while the blue dots represent the significantly downregulated genes. **(B)** Venn image of arDEGs in NEC. **(C)** A heatmap of the 47 arDEGs in NEC and CTRL mice. **(D)** The boxplot of 47 arDEGs in NEC and CTRL groups. **(E)** The protein–protein interaction (PPI) analysis of the 47 arDEGs. **(F)** Gene Ontology (GO) enrichment analysis of 47 arDEGs. **(G)** Kyoto Encyclopedia of Genes and Genomes (KEGG) enrichment signaling pathway analysis of 47 arDEGs.

Illumina sequencing identified messenger RNAs (mRNAs) in neonatal NEC and CTRL mouse intestinal tissues were identified. The volcano plot showed the distribution of DEGs [*p* < 0.05 and absolute value of log2 (fold change) ≥1] (Figure 2A). To explore the autophagy-related genes expressed in NEC, 222 autophagy-related genes were collected altogether from the Human Autophagy Database. Venn diagrams revealed 47 arDEGs in NEC (Figure 2B), including 34 upregulated and 13 downregulated genes (Table 1). Next, 47 arDEGs between NEC and CTRL groups were obtained and performed in a heatmap *via* R software (Figure 2C). Furthermore, the expression patterns of the above 47 genes between NEC and CTRL groups were shown by box plots (Figure 2D). The top five upregulated genes were ITGA6, RAC1, PTK6, ATG9B, and MAP2K7, and the top five downregulated genes were MAPK8IP1, TSC1, RPS6KB1, SAR1A, and FKBP1B (Figure 2D and Table 1).

**Table 1:** The 47 differentially expressed autophagy-related genes in experimental necrotizing enterocolitis (NEC).

**Gene**	**Log2FC**	***P*-value**	**Changes**
ITGA6	5.770033	7.99E−06	Up
RAC1	4.508116	0.001477	Up
PTK6	3.380483	7.67E−09	Up
ATG9B	3.368293	3.07E−07	Up
MAP2K7	3.164058	0.005586	Up
NBR1	3.114138	0.018878	Up
IKBKB	3.017975	0.007636	Up
FOS	3.002119	3.15E−06	Up
CFLAR	2.745846	0.000623	Up
CLN3	2.640714	8.07E−05	Up
SH3GLB1	2.27457	0.000181	Up
BAK1	2.137084	0.006199	Up
BECN1	2.084638	0.002926	Up
PIK3C3	2.076903	0.006382	Up
VEGFA	1.93119	0.000418	Up
EIF4G1	1.852582	5.26E−06	Up
ITGB4	1.780454	0.044816	Up
BNIP1	1.457028	0.024704	Up
ATG9A	1.430786	0.002751	Up
RPTOR	1.401169	9.49E−05	Up
EIF2AK2	1.304574	0.019495	Up
PRKCD	1.257852	0.003734	Up
HIF1A	1.233775	0.005132	Up
NCKAP1	1.21286	0.000286	Up
CDKN1A	1.210085	0.00461	Up
ATG7	1.161993	0.042873	Up
AMBRA1	1.101969	0.034702	Up
HSPA5	1.091576	0.001004	Up
UVRAG	1.07988	0.027448	Up
ITGA3	1.069406	0.003999	Up
SIRT2	1.063647	0.003214	Up
ERO1L	1.026953	0.003651	Up
NAMPT	1.022692	0.002057	Up
HDAC1	1.011497	0.044572	Up
TM9SF1	−1.40332	0.033662	Down
BCL2	−1.59	0.020196	Down
DLC1	−1.59468	0.035385	Down
ITPR1	−1.67036	0.016809	Down
DAPK1	−1.68858	0.005928	Down
NRG2	−1.89838	0.018238	Down
RAB11A	−2.02974	0.006036	Down
ATG16L2	−2.1291	0.042323	Down
FKBP1B	−2.30953	0.005757	Down
SAR1A	−2.57663	0.034802	Down
RPS6KB1	−3.00558	0.022846	Down
TSC1	−3.28312	0.004162	Down
MAPK8IP1	−3.5585	0.000348	Down

### PPI Network, GO, and KEGG Enrichment Analysis of the arDEGs in a Mouse Model of NEC

To reveal the interactions among arDEGs, PPI analysis was carried out, and the PPI network is shown in Figure 2E, demonstrating those autophagy-related genes interplayed with one another. To further find the potential biological and molecular functions of the above arDEGs, GO and KEGG enrichment analyses were performed and the results illustrated that the most significant GO enriched terms included the process of autophagic mechanism, autophagy, and macroautophagy (BP); autophagosome, late endosome, and phagophore assembly site (CC); protein serine/threonine kinase activity, ubiquitin-like protein ligase binding, and ubiquitin-protein ligase binding (MF) (Figure 2F). In addition, in KEGG enrichment results, the above genes are primarily involved in the process of autophagy, Kaposi sarcoma-associated herpesvirus infection, mitophagy, and the PI3K-Akt signaling pathway (Figure 2G).

### Identification and Analysis of Autophagy-Related Genes in Human NEC Through Analyzing the GSE46619 Dataset From the GEO Database

**Figure 3:**
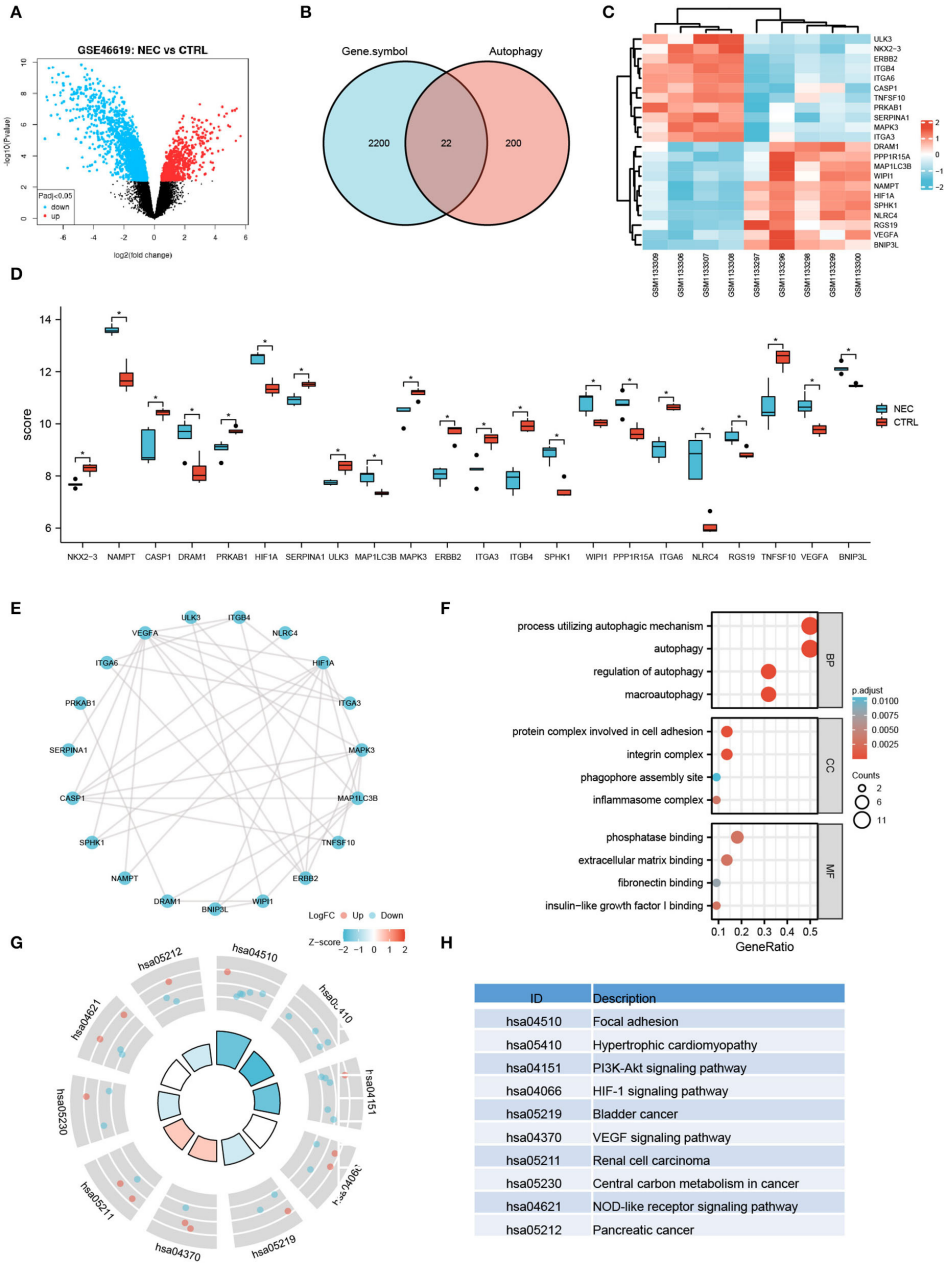
Autophagy-related differentially expressed genes in human NEC from dataset GSE46619. **(A)** Volcano plot of the DEGs in human NEC and CTRL samples from GSE46619. The red dots represent the significantly upregulated genes and the blue dots indicate the significantly downregulated genes. **(B)** Venn image of arDEGs. **(C)** A heatmap of the 22 arDEGs in human NEC and CTRL tissues. **(D)** The boxplot of 22 arDEGs in human NEC and CTRL tissues. **(E)** The PPI analysis of the 22 arDEGs. **(F)** Gene Ontology enrichment analysis of 22 arDEGs. **(G,H)** Kyoto Encyclopedia of Genes and Genomes enrichment signaling pathway analysis of 22 arDEGs. *p < 0.05.

To further investigate the autophagy-related genes in NEC, we identified the arDEGs of NEC by analyzing the dataset GSE46619 from the GEO database. In total, 133 DEGs were found (Figure 3A), and 22 autophagy-related genes were determined by using the Venn analysis (Figure 3B), including 11 upregulated and 11 downregulated genes (Table 2). The 22 arDEGs between NEC and CTRL groups were performed in a heatmap figure *via* R software (Figure 3C). Additionally, the expression patterns of 22 arDEGs between NEC and CTRL groups were revealed by box plots (Figure 3D). The top 5 upregulated genes consisted of NLRC4, NAMPT, SPHK1, DRAM1, and HIF1A, and the top 5 downregulated genes consisted of ITGB4, TNFSF10, ERBB2, ITGA6, and CASP1 (Figure 3D and Table 2).

**Table 2:** The 22 differentially expressed autophagy-related genes in human NEC derived from dataset GSE46619.

**Gene**	**Log2FC**	**P-value**	**Changes**
NLRC4	2.581	2.89E−05	Up
NAMPT	1.84	1.28E−05	Up
SPHK1	1.418	5.32E−05	Up
DRAM1	1.352	0.003162	Up
HIF1A	1.157	6.34E−05	Up
PPP1R15A	1.119	0.00063	Up
VEGFA	0.927	0.001282	Up
MAP1LC3B	0.795	0.00027	Up
WIPI1	0.792	0.003459	Up
BNIP3L	0.674	0.000467	Up
RGS19	0.657	0.003373	Up
NKX2-3	−0.583	0.002178	Down
SERPINA1	−0.601	0.001858	Down
ULK3	−0.615	0.001626	Down
PRKAB1	−0.698	0.002698	Down
MAPK3	−0.761	0.002692	Down
ITGA3	−1.155	0.000728	Down
CASP1	−1.311	0.001702	Down
ITGA6	−1.608	1.48E−05	Down
ERBB2	−1.636	1.06E−05	Down
TNFSF10	−1.833	0.000508	Down
ITGB4	−2.091	4.36E−06	Down

The PPI analysis illustrated the interaction among those autophagy-related genes with one another (Figure 3E). GO enrichment analysis displayed that the most significant GO enriched terms involved in the process of autophagic mechanism, autophagy, and regulation of autophagy (BP); protein complex involved in cell adhesion, integrin complex, and phagophore assembly site (CC); phosphatase binding, extracellular matrix binding, and fibronectin-binding (MF) (Figure 3F). In KEGG enrichment analysis, the arDEGs were mainly contained in the processes of focal adhesion, hypertrophic cardiomyopathy, PI3K-Akt signaling pathway, and HIF-1 signaling pathway (Figures 3G,H).

### Validation of Autophagy-Related Genes in a Mouse Model of NEC

**Figure 4:**
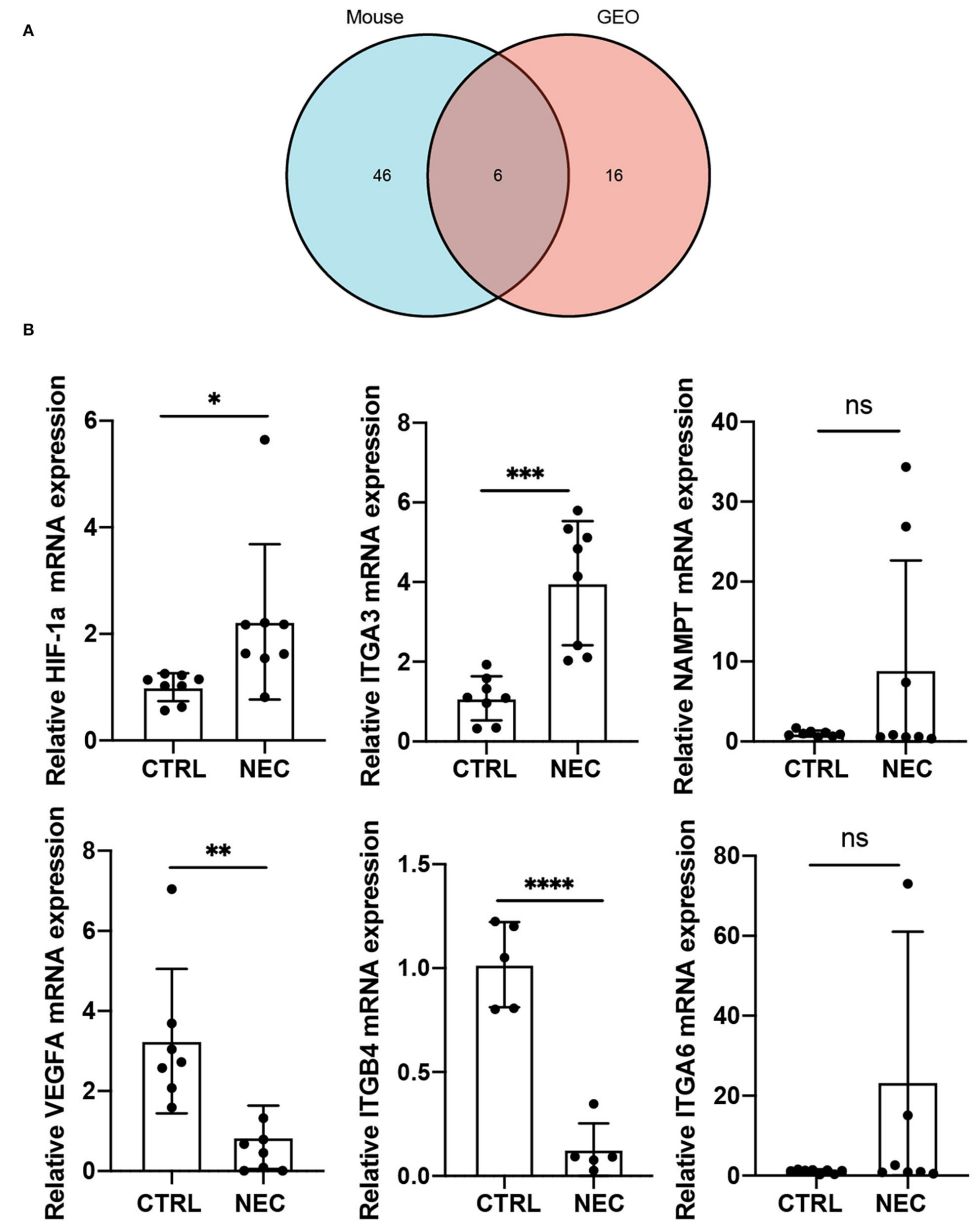
Validation of co-expressed autophagy-4elated genes in a mouse model of NEC. **(A)** Venn image of co-expressed autophagy-related genes in a mouse model of NEC and human NEC from dataset GSE46619. **(B)** The mRNA expression level of co-expressed autophagy-related genes in a mouse model of NEC. ns, no significance; *p* > 0.05; *p < 0.05; **p < 0.01; ***p < 0.001; and ****p < 0.0001.

To further investigate autophagy-related genes in NEC, we analyzed the arDEGs derived from the previous analysis in our neonatal mouse model and dataset GSE46619 *via* Venn analysis. Six autophagy-related genes were expressed in the two datasets that included HIF-1a, VEGFA, ITGA3, ITGA6, NAMPT, and ITGB4 (Figure 4A). The expression levels of the six autophagy-related genes were further evaluated *via* qRT-PCR in the mouse model of NEC. Similar to the results of RNA-sequencing in the mouse model of NEC, the mRNA levels of HIF-1a and ITGA3 were significantly higher in the NEC group than CTRL group (Figure 4B). However, the expression levels of VEGFA and ITGB4 were significantly lower in the NEC group (Figure 4B). In addition, the mRNA levels of NAMPT and ITGA6 demonstrated no significant statistical difference between the two groups (Figure 4B).

## Discussion

In the present study, we find that autophagy was upregulated in necrotizing enterocolitis. Besides, six autophagy-related genes were determined both differentially expressed in the mouse model and dataset GSE46619. Furthermore, further validation was performed by qRT-PCR in the mouse model of NEC, and HIF-1a and ITGA3 were upregulated significantly in NEC.

In this study, we established an experimental NEC mouse model through the combination of oral feeding of hyperosmolar formula milk, exposure to hypoxia, and LPS, which is the same as the methods of previous studies by Dr. Pierro's lab (18, 19). Similar to their results, the experimental NEC mouse presented obvious NEC-like intestinal damage and poor nutritional status. In Dr. Hackam's studies, the NEC model was constructed in 7- to 8-day-old C57BL/6 pups by feeding enteral formula, hypoxia (5% O_2_), and administration of enteric bacteria from a patient with NEC, resulting in patchy necrosis and inflammation in the ileum that closely resembles clinical NEC injury (20, 21).

Growing evidence indicates that autophagy refers to the progression of NEC (22–25). Autophagy was activated both in the intestinal epithelium of patients with NEC and in the ileum of a rat model of NEC. Autophagy was required for the development of NEC, not only as a result of NEC (22). Moreover, Human β-defensin-3 (hBD3) reduces excessive autophagy in intestinal epithelial cells and NEC. Furthermore, hBD3 treatment can significantly induce intestinal epithelial cell migration and reduce the severity and mortality of the NEC model in neonatal rats (16). Similarly, erythropoietin (Epo), a component of breast milk, protects intestinal epithelium from excessive autophagy and apoptosis in NEC mice (24). In a rat model of NEC, EGF treatment also reduced NEC injury by the regulation of intestinal autophagy (22, 26). These studies all indicated that the modulation of autophagy can be considered a potential therapeutic approach for NEC.

Autophagy is critically implicated in various fundamental cellular processes. Therefore, the challenge of therapeutic targeting of autophagy is to modulate autophagy without adversely affecting other cellular processes. Multiple clinical trials testing the efficacy of autophagy inhibitors, chloroquine (CQ), and hydroxychloroquine (HCQ), have been conducted in cancer therapy and other diseases. However, the results are largely disappointing. Moreover, there is no available drug specific targeting autophagy for NEC. Autophagy inhibitors combined with specific therapeutic targets of autophagy may be a better strategy for NEC therapy. Thus, further studies need to be focused on the role and regulatory mechanism of autophagy-related proteins in NEC development.

In our study, we identified 47 arDEGs in the neonatal mouse model of NEC and 22 arDEGs in dataset GSE46619. The GO and KEGG enrichment analysis of arDEGs both displayed several enriched terms related to autophagy and macroautophagy. We further identified 6 genes both differentially expressed in our mouse model and dataset GSE46619, and validated them in a mouse model of NEC. Hypoxia can induce autophagy in a HIF-1a-dependent pathway in various research, and HIF-1a is a major transcription factor related to hypoxia (27–29). Hypoxia and oxidative stress have been confirmed as vital factors in the progression of NEC (28, 30). In the present study, we found the expression level of HIF-1a increased in NEC. A previous report also revealed that serum HIF-1a was raised in patents with NEC (25). Moreover, HIF-1a is involved in the disease severity of NEC in animal models (31). Thus, as an autophagy-related gene, HIF-1a plays a vital role in the progression of NEC. ITGA3 belongs to the integrin family of cell surface receptors and is related to autophagy in various cancers, such as head and neck squamous cell carcinoma, bladder cancer, osteosarcoma, and glioblastoma (32–35).

There are some limitations to our research. First, the quantity of human NEC samples included in the GEO dataset is finite, and we need to perform experimental verification in a larger NEC cohort of clinical samples in future studies. Second, we only evaluated the expression level of the arDEGs in the mouse model of NEC but did not investigate the deep mechanism and function of these genes in the progression of NEC.

In summary, our findings support that the autophagy process involves the NEC of both neonatal mice and the human intestine, and therefore, our research enhances the grasp of the relationship between neonatal NEC progression and autophagy.

## Data Availability Statement

The original contributions presented in the study are publicly available. This data can be found at: https://www.ncbi.nlm.nih.gov/geo/query/acc.cgi?acc=GSE198372.

## Ethics Statement

The animal study was reviewed and approved by Children's Hospital of Fudan University.

## Author Contributions

YT designed and performed the experiments, analyzed data, and wrote the manuscript. MM and XC provided partial experimental animal tissue and performed the experiments. CS and HZ provided critical suggestions and financial support. All authors read and approved the final manuscript.

## Funding

This study was supported by grants from the National Natural Science Foundation of China (8187060810).

## Conflict of Interest

The authors declare that the research was conducted in the absence of any commercial or financial relationships that could be construed as a potential conflict of interest.

## Publisher's Note

All claims expressed in this article are solely those of the authors and do not necessarily represent those of their affiliated organizations, or those of the publisher, the editors and the reviewers. Any product that may be evaluated in this article, or claim that may be made by its manufacturer, is not guaranteed or endorsed by the publisher.

## References

[1] Frost BL Caplan MS. Probiotics and prevention of neonatal necrotizing enterocolitis. Curr Opin Pediatr. (2011) 23:151–5. 10.1097/MOP.0b013e328343d65f 21252675

[2] Salhab WA Perlman JM Silver L Sue Broyles R. Necrotizing enterocolitis and neurodevelopmental outcome in extremely low birth weight infants <1000 g. J Perinatol. (2004) 24:534–40. 10.1038/sj.jp.7211165 15254558

[3] Hintz SR Kendrick DE Stoll BJ Vohr BR Fanaroff AA Donovan EF. Neurodevelopmental and growth outcomes of extremely low birth weight infants after necrotizing enterocolitis. Pediatrics. (2005) 115:696–703. 10.1542/peds.2004-0569 15741374

[4] Martin CR Walker WA. Probiotics: role in pathophysiology and prevention in necrotizing enterocolitis. Semin Perinatol. (2008) 32:127–37. 10.1053/j.semperi.2008.01.006 18346537

[5] Parzych KR Klionsky DJ. An overview of autophagy: morphology, mechanism, and regulation. Antioxid Redox Signal. (2014) 20:460–73. 10.1089/ars.2013.5371 23725295 PMC3894687

[6] Cao W Li J Yang K Cao D. An overview of autophagy: mechanism, regulation and research progress. Bull Cancer. (2021) 108:304–22. 10.1016/j.bulcan.2020.11.004 33423775

[7] Jiang P Mizushima N. Autophagy and human diseases. Cell Res. (2014) 24:69–79. 10.1038/cr.2013.161 24323045 PMC3879707

[8] Dikic I Elazar Z. Mechanism and medical implications of mammalian autophagy. Nat Rev Mol Cell Biol. (2018) 19:349–64. 10.1038/s41580-018-0003-4 29618831

[9] Deretic V Saitoh T Akira S. Autophagy in infection, inflammation and immunity. Nat Rev Immunol. (2013) 13:722–37. 10.1038/nri3532 24064518 PMC5340150

[10] Russell RC Yuan HX Guan KL. Autophagy regulation by nutrient signaling. Cell Res. (2014) 24:42–57. 10.1038/cr.2013.166 24343578 PMC3879708

[11] Cadwell K. Crosstalk between autophagy and inflammatory signalling pathways: balancing defence and homeostasis. Nat Rev Immunol. (2016) 16:661–75. 10.1038/nri.2016.100 27694913 PMC5343289

[12] Matsuzawa-Ishimoto Y Hwang S Cadwell K. Autophagy and Inflammation. Annu Rev Immunol. (2018) 36:73–101. 10.1146/annurev-immunol-042617-053253 29144836

[13] Deretic V. Autophagy in inflammation, infection, and immunometabolism. Immunity. (2021) 54:437–53. 10.1016/j.immuni.2021.01.018 33691134 PMC8026106

[14] Xia H Green DR Zou W. Autophagy in tumour immunity and therapy. Nat Rev Cancer. (2021) 21:281–97. 10.1038/s41568-021-00344-2 33758415 PMC8087647

[15] Bento CF Renna M Ghislat G Puri C Ashkenazi A Vicinanza M. Mammalian autophagy: how does it work? Annu Rev Biochem. (2016) 85:685–713. 10.1146/annurev-biochem-060815-014556 26865532

[16] Chen L Lv Z Gao Z Ge G Wang X Zhou J. Human beta-defensin-3 reduces excessive autophagy in intestinal epithelial cells and in experimental necrotizing enterocolitis. Sci Rep. (2019) 9:19890. 10.1038/s41598-019-56535-3 31882811 PMC6934505

[17] Zani A Cordischi L Cananzi M De Coppi P Smith VV Eaton S. Assessment of a neonatal rat model of necrotizing enterocolitis. Eur J Pediatr Surg. (2008) 18:423–6. 10.1055/s-2008-1038951 19012230

[18] Li B Lee C Cadete M Zhu H Koike Y Hock A. Impaired Wnt/β-catenin pathway leads to dysfunction of intestinal regeneration during necrotizing enterocolitis. Cell Death Dis. (2019) 10:743. 10.1038/s41419-019-1987-1 31582728 PMC6776513

[19] Li B Lee C O'Connell JS Antounians L Ganji N Alganabi M. Activation of Wnt signaling by amniotic fluid stem cell-derived extracellular vesicles attenuates intestinal injury in experimental necrotizing enterocolitis. Cell Death Dis. (2020) 11:750. 10.1038/s41419-020-02964-2 32929076 PMC7490270

[20] Leaphart CL Cavallo J Gribar SC Cetin S Li J Branca MF. A critical role for TLR4 in the pathogenesis of necrotizing enterocolitis by modulating intestinal injury and repair. J Immunol. (2007) 179:4808–20. 10.4049/jimmunol.179.7.4808 17878380

[21] Lu P Yamaguchi Y Fulton WB Wang S Zhou Q Jia H. Maternal aryl hydrocarbon receptor activation protects newborns against necrotizing enterocolitis. Nat Commun. (2021) 12:1042. 10.1038/s41467-021-21356-4 33589625 PMC7884836

[22] Maynard AA Dvorak K Khailova L Dobrenen H Arganbright KM Halpern MD. Epidermal growth factor reduces autophagy in intestinal epithelium and in the rat model of necrotizing enterocolitis. Am J Physiol Gastrointest Liver Physiol. (2010) 299:G614–622. 10.1152/ajpgi.00076.2010 20539009 PMC2950687

[23] Neal MD Sodhi CP Dyer M Craig BT Good M Jia H. A critical role for TLR4 induction of autophagy in the regulation of enterocyte migration and the pathogenesis of necrotizing enterocolitis. J Immunol. (2013) 190:3541–51. 10.4049/jimmunol.1202264 23455503 PMC3608826

[24] Yu Y Shiou SR Guo Y Lu L Westerhoff M Sun J. Erythropoietin protects epithelial cells from excessive autophagy and apoptosis in experimental neonatal necrotizing enterocolitis. PLoS ONE. (2013) 8:e69620. 10.1371/journal.pone.0069620 23936061 PMC3723879

[25] Yuan Y Ding D Zhang N Xia Z Wang J Yang H. TNF-alpha induces autophagy through ERK1/2 pathway to regulate apoptosis in neonatal necrotizing enterocolitis model cells IEC-6. Cell Cycle. (2018) 17:1390–402. 10.1080/15384101.2018.1482150 29950141 PMC6110600

[26] Coursodon CF Dvorak B. Epidermal growth factor and necrotizing enterocolitis. Curr Opin Pediatr. (2012) 24:160–4. 10.1097/MOP.0b013e3283504ddb 22227788

[27] Fang Y Tan J Zhang Q. Signaling pathways and mechanisms of hypoxia-induced autophagy in the animal cells. Cell Biol Int. (2015) 39:891–8. 10.1002/cbin.10463 25808799

[28] Choi H Merceron C Mangiavini L Seifert EL Schipani E Shapiro IM. Hypoxia promotes noncanonical autophagy in nucleus pulposus cells independent of MTOR and HIF1A signaling. Autophagy. (2016) 12:1631–46. 10.1080/15548627.2016.1192753 27314664 PMC5082780

[29] Liu C Li S Pang F Wu H Chai L Liang C. Autophagy-related gene expression regulated by HIF-1alpha in salivary adenoid cystic carcinoma. Oral Dis. (2019) 25:1076–83. 10.1111/odi.13058 30746817

[30] Qureshi-Baig K Kuhn D Viry E Pozdeev VI Schmitz M Rodriguez F. Hypoxia-induced autophagy drives colorectal cancer initiation and progression by activating the PRKC/PKC-EZR (ezrin) pathway. Autophagy. (2020) 16:1436–52. 10.1080/15548627.2019.1687213 31775562 PMC7469473

[31] Bai M Lu C An L Gao Q Xie W Miao F. SIRT1 relieves necrotizing enterocolitis through inactivation of Hypoxia-inducible factor (HIF)-1a. Cell Cycle. (2020) 19:2018–27. 10.1080/15384101.2020.1788251 32657204 PMC7469541

[32] Yang C Mei H Peng L Jiang F Xie B Li J. Prognostic Correlation of an Autophagy-Related Gene Signature in Patients with Head and Neck Squamous Cell Carcinoma. Comput Math Methods Med. (2020) 2020:7397132. 10.1155/2020/7397132 33456497 PMC7785385

[33] Qi W Yan Q Lv M Song D Wang X Tian K. Prognostic signature of osteosarcoma based on 14 autophagy-related genes. Pathol Oncol Res. (2021) 27:1609782. 10.3389/pore.2021.1609782 34335109 PMC8322075

[34] Ren Z Zhang L Ding W Luo Y Shi Z Shrestha B. Development and validation of a novel survival model for head and neck squamous cell carcinoma based on autophagy-related genes. Genomics. (2021) 113:1166–75. 10.1016/j.ygeno.2020.11.017 33227411

[35] Zhou C. Li A.H. Liu S. Sun H. (2021). Identification of an 11-autophagy-related-gene signature as promising prognostic biomarker for bladder cancer patients. Biology (Basel). (2021) 10:375. 10.3390/biology10050375 33925460 PMC8146553

