# Supplementary material for: Identification and Validation of Autophagy-Related Genes in Necrotizing Enterocolitis
Source: Front Pediatr. 2022 Apr 28;10:839110. doi: 10.3389/fped.2022.839110 (PMC9096030; doi:10.3389/fped.2022.839110)
Supplement: Supplementary file 1 [file Table_1.DOCX]

Supplementary Material

# Supplementary Data

| **Gene** | **Species** | **Forward sequence** | **Reverse sequence** |
| --- | --- | --- | --- |
| GAPDH | Mouse | TGACCTCAACTACATGGTCTACA | CTTCCCATTCTCGGCCTTG |
| IL-6 | Mouse | TAGTCCTTCCTACCCCAATTTCC | TTGGTCCTTAGCCACTCCTTC |
| Lgr5 | Mouse | GGACCAGATGCGATACCGC | CAGAGGCGATGTAGGAGACTG |
| ITGA6 | Mouse | GAGACTGGAGTTTCTGCGATG | TTCTACACGGACGATCCCTTT |
| VEGFA | Mouse | GCACATAGAGAGAATGAGCTTCC | CTCCGCTCTGAACAAGGCT |
| NAMPT | Mouse | GCAGAAGCCGAGTTCAACATC | TTTTCACGGCATTCAAAGTAGGA |
| ITGA3 | Mouse | GTGTGACTTCTTTAAGCCGACC | GATACGCACTGCATGGTACTT |
| HIF-1a | Mouse | ACCTTCATCGGAAACTCCAAAG | CTGTTAGGCTGGGAAAAGTTAGG |
| ITGB4 | Mouse | AGAGCTGTACCGAGTGCATC | TGGTGTCGATCTGGGTGTTCT |

**Supplementary Table 1.** Primer Sequences for Quantitative Reverse Transcriptase Polymerase Chain Reaction.
